# Novel Methodologies for Multiaxial Strain Measurements with Piezoresistive Films based on Graphene Nanoplatelets

**DOI:** 10.1002/smsc.202100088

**Published:** 2021-10-24

**Authors:** Volkan Yokaribas, Peter Kraemer, Alexander B. Mende, Jasper Ruhkopf, Max C. Lemme, Claus-Peter Fritzen

**Affiliations:** ^1^ Department of Mechanical Engineering University of Siegen 57076 Siegen Germany; ^2^ Advanced Microelectronic Center Aachen (AMICA) AMO GmbH 52074 Aachen Germany; ^3^ Chair of Electronic Devices RWTH Aachen University 52074 Aachen Germany

**Keywords:** graphene nanoplatelets, piezoresistive effect, spray deposition, strain-differential electrical impedance tomography

## Abstract

Many recent investigations in the context of graphene nanoplatelets (GNPs) coatings report surface strain measurements by using piezoresistive sensing capabilities. An often underestimated problem is that the strain field is unknown and the principal strain components as well as their orientations must be determined. Herein, GNP films subjected to multiaxial strain are examined. Experimental results show that although the sensitivity to longitudinal strain is the highest, the ratio between transverse and longitudinal sensitivity exceeds 0.5. The sensitivity to shear strain is much lower. A model assisted study of a random network provides additional guidelines for the different electromechanical sensitivities. In practice, the GNP film is usually subjected to different strains simultaneously so that the multiaxial strain measurement becomes difficult. Therefore, two novel approaches for sensing plane strain components with circular GNP films are developed and successfully verified in experiments. The numerical approach is called strain‐differential electrical impedance tomography (SD‐EIT), where the proposed piezoresistive model elementwise in a finite element model is implemented and the strain components of a strain rosette are reconstructed. Moreover, an analytical approach is derived from SD‐EIT and exhibits further the opportunity to detect anomalies within the piezoresistive sensing behavior of GNP films.

## Graphical abstract

Graphene nanoplatelets (GNPs) films promote the formation of conductive paths with piezoresistive sensing capabilities. Herein, how the resistance of GNP film is affected by multiaxial strain is theoretically and experimentally examined. To accurately characterize the strain field with GNP films, novel methodologies are presented. The goal is to determine the principal strains in an unknown 2D strain field.

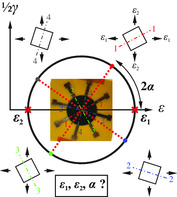

## 1 Introduction

Various low‐cost technologies (e.g., ink‐jet printing^[^ ^1^ ^]^ and spray coating^[^ ^2^, ^3^ ^]^) to deposit graphene nanoplatelets (GNP) within dispersion or inks on surfaces are already known and successfully implemented. The formation of GNP films provides multifunctional sensing capabilities in terms of various external stimuli (e.g., temperature^[^ ^4^ ^]^ and humidity^[^ ^5^ ^]^), since the conductivities of these films are affected. Due to the piezoresistive effect (PRE) of GNP films, various applications associated with strain measurements are envisaged for future technologies, such as human motion and gesture recognition or tactile sensation in electronic skins.^[^ ^6^ ^]^ The implementation of such films also provides the path for highly flexible and sensitive strain gauges for static and dynamic loadings.^[^ ^7^ ^]^ In previous works^[^ ^8^, ^9^ ^]^ spray‐coated carbon films were already investigated to measure spatial strains with the attributed PRE. A common way is to measure strain at a specific point or along a path with traditional strain measurement devices (e.g., metallic,^[^ ^10^ ^]^ semiconductive strain gauges,^[^ ^11^ ^]^ or fiber Bragg gratings^[^ ^12^ ^]^). Advantages of strain measurements with carbon allotrope films are seen in the low‐cost deposition technique, spatial strain sensing capabilities^[^ ^13^ ^]^ and the higher electromechanical sensitivity,^[^ ^14^ ^]^ which is a crucial point for small strain detection. The PRE of films based on GNP is attributed to changes of contact resistances between adjacent particles due to changes of overlapping areas or tunneling effect.^[^ ^15^ ^]^ Recently published papers characterize the electromechanical response of printed nanomaterial films with a single gauge factor and use rosettes of these films for multiaxial strain sensing in a wrong way.^[^ ^16^ ^]^ A tensorial description of the PRE effect of carbon nanotube (CNT) films is published by Zhao.^[^ ^13^ ^]^ This approach consists of the laws for describing the PRE for semiconductors with cubic single crystal structure from Smith.^[^ ^17^ ^]^ The theory of Smith was transferred with its simplifications of cubic symmetry to characterize the PRE of a random network of CNT films.^[^ ^9^, ^13^ ^]^ In this article, an empirical piezoresistive model is introduced for multiaxial strain sensing of GNP films considering the direction of current flow and the strain components corresponding to that axis. Derived from the research of polycrystalline metals from Bridgman^[^ ^10^ ^]^ and the work for semiconducting materials from Pfann and Thungston,^[^ ^18^ ^]^ it is generally assumed that the relative resistance change $\frac{\Delta R}{R}$ in a gauge axis of a film is affected by all surface strain components. In addition to experimental investigations, where the GNP film is solely subjected to uniaxial normal strain or shear strain, we validate our results in a model based approach. First, a 3D architecture of the GNP network is modeled, where the GNP are parallel aligned to substrate's surface and the contact resistances are mostly dominated by overlapping areas of adjacent particles. To consider a random network, which is mainly affected by changes in tunneling distances due to in‐plane deformation, a simplified model in 2D architecture is considered. We assume that while the substrate is subjected to longitudinal, transverse, or shear strain, respectively, the GNP particles undergo a rigid body motion related to the deformation state. The resistance changes are correspondently expressed by changes in overlapping areas or tunneling distances. The deformed state of the network is related to the initial (undeformed) state, which is kept equal for longitudinal, transverse, and shear sensitivity calculation. Our goal is to determine the related principal strains once the GNP film is subjected to an unknown 2D strain field.

We introduce the strain‐differential electrical impedance tomography (SD‐EIT), where we implement the empirical piezoresistive model elementwise in a finite element model and reconstruct the strain components of a delta rosette using the change of measured signal. Additionally, we also develop an analytical approach derived from SD‐EIT. Here, the principal strain axes in an unknown strain field are calculated by at least three integrated gauge axes within a GNP film. For validation purposes a fourth gauge axis is used. All the experimental investigations are done using isotropic substrate materials with homogenous strain distribution so that strain components can accurately be expressed according to Mohr's strain circle. All studies are based on infinitesimal strain theory with an envisaged application in the field of non‐destructive testing or condition monitoring of engineering structures.

## 2 Electromechanical Sensitivities of GNP film

### 2.1 Preliminary Investigations

**Figure 1:**
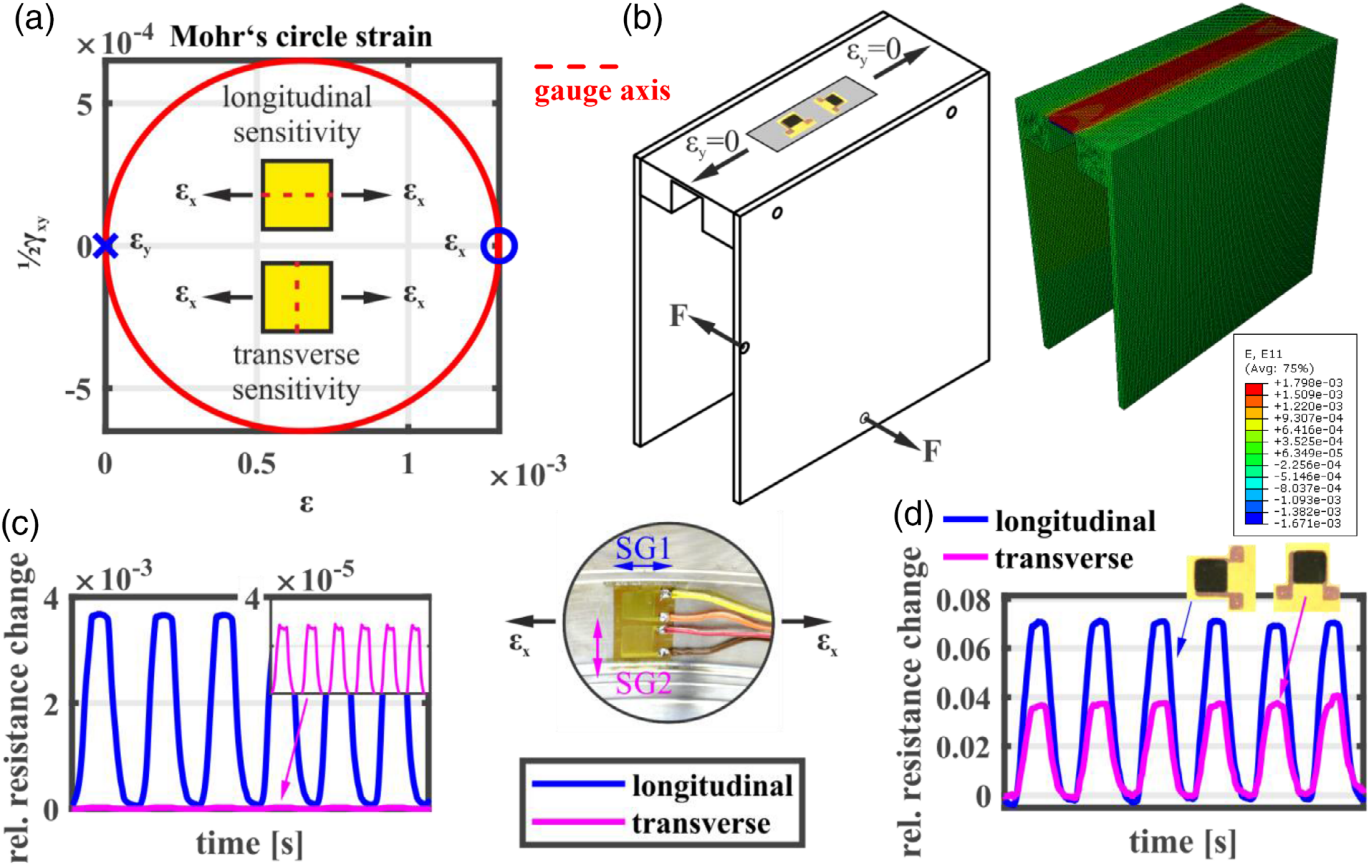
a) Mohr's circle representation of the uniaxial strain *ε*_*x*_ and determination of the longitudinal and transverse sensitivity according to gauge axis alignment. b) Specimen subjected to uniaxial strain *ε*_*x*_ and validated in a finite element analysis. c) Signal of SG1 (strain axis aligned to gauge axis) and SG2 (strain axis transversely aligned to gauge axis). d) Signal of two GNP films, where the current flow direction is subjected to longitudinal strain or transverse strain.

Metallic strain gauges are optimized (e.g., grid design and gauge construction) to measure uniaxial strain components in sensing axis without considering transverse strain and shear strain sensitivity at a specific point, either.^[^ ^19^ ^]^ Hence, a metallic strain gauge is often described by a single gauge factor $\text{GF}=\Delta R/R\cdot \varepsilon_{L}$, related to longitudinal strain *ε*_L_ in gauge axis direction.^[^ ^20^ ^]^ The transverse sensitivity is investigated using a conventional test specimen,^[^ ^21^ ^]^ where the gauge axis is merely elongated in uniaxial strain direction *ε*_x_ in a nearly homogenous strain field (see finite element analysis in **Figure** **1**b). Once the strain gauge (SG) is perpendicularly attached to that axis the transverse sensitivity is measured. We apply two metallic SGs, where SG1 is attached parallel to the strain axis *ε*_*x*_ and SG2 perpendicular to that axis (see Figure 1c). The results in Figure 1c show that the signal of SG1 is two orders higher than SG2, so that the transverse sensitivity is often negligible in practice.^[^ ^18^ ^]^ The small transverse sensitivity of metallic SG is attributed to small errors in gauge construction.^[^ ^19^ ^]^ In comparison to that the electromechanical response of a GNP film shows a higher transverse sensitivity, as exemplary investigated for two perpendicularly attached GNP films in Figure 1d. The longitudinal measurement denotes the direction, where the current flow from one to second electrode and the strain axis is in the same direction. In this context, the GNP is macroscopically seen as a homogenous electrical conductor, which is attributed to a random distribution of GNP within the film. The characterization of the same specimen in its longitudinal and transverse sensitivity is not possible since additional electrodes must be attached with limited space to existing electrodes. Therefore, we attach a second GNP film, which is produced in the same production batch using our home‐made spraying system,^[^ ^22^ ^]^ so that similar piezoresistive characteristics are assumed. The experimentally determined longitudinal strain sensitivities of five different GNP films show a linear electromechanical behavior in all cases (see Section 3, Supporting Information). The results also exhibit higher longitudinal than transverse sensitivities, which can be explained by geometry. The substrate's deformation field shows a both‐sided elongation (in the case of tensile stress), where a midline without any deformation exists. The lower transverse sensitivity for the same network is examined since this midline is parallel to current flow direction in the case of transverse strain and results in lower overall change of resistance.

**Figure 2:**
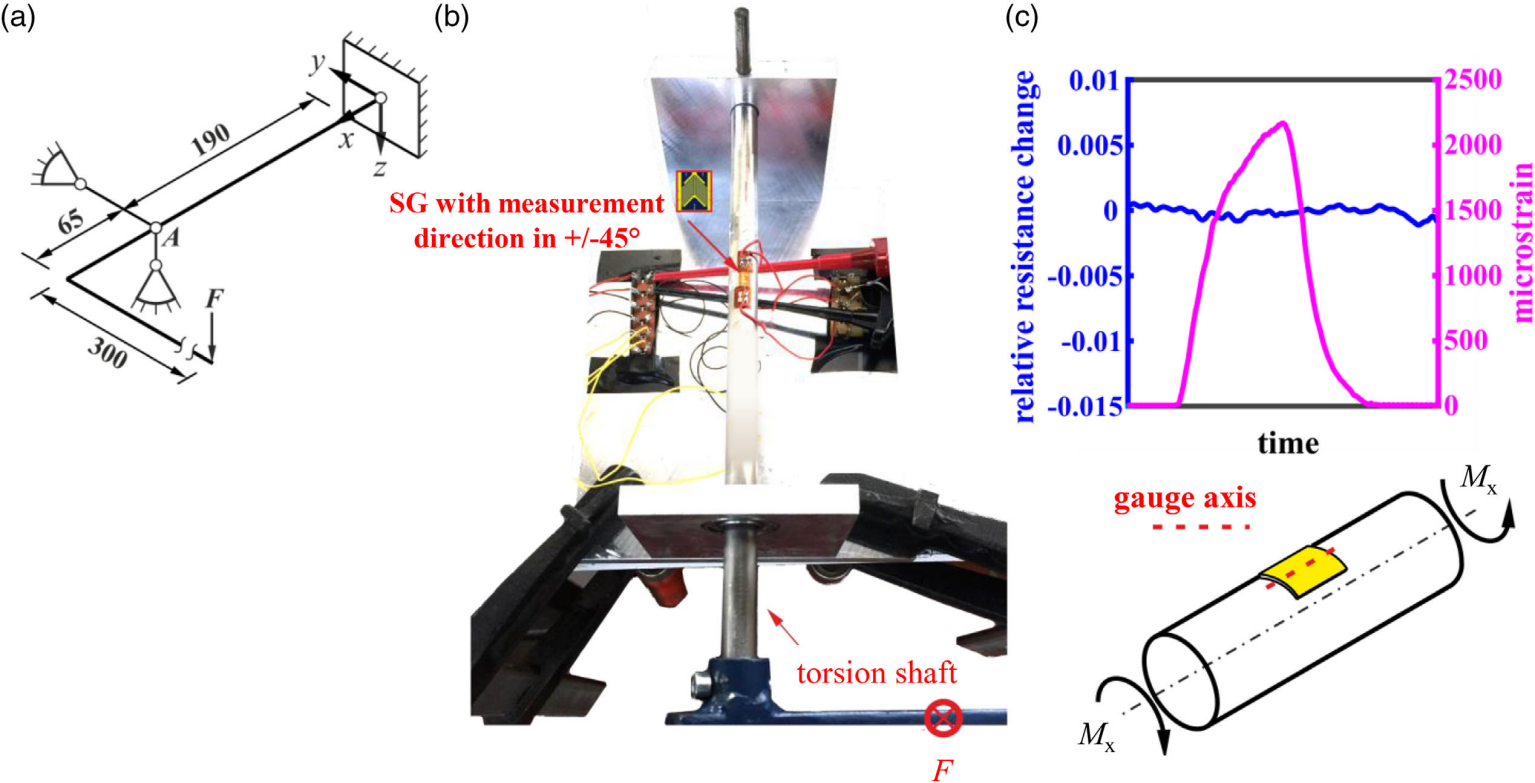
a) Uniaxial strain field in Mohr's circle representation for a cylindrical shaft loaded by a torque (principal strain of 2160 microstrain). b) Mechanical device in experiment. c) Signal of V‐shaped SG and GNP film during shear strain loading.

By definition, the relation of the aforementioned strain components to the resistance change shall be considered by the longitudinal piezoresistive effect *k*_L_ (l‐PRE) and the transverse piezoresistive effect *k*_T_ (t‐PRE). Once the gauge axis is not aligned to the principal strain direction, shear may also affect the corresponding gauge line. In this work, the piezoresistive shear effect *k*_S_ (s‐PRE) is defined by the relation between an engineering shear strain *ε*_*xy*_ *= γ*_*xy*_/2 and its contribution to measured resistance in gauge axis, which equals the aforementioned direction of current flow. Since we are interested in the s‐PRE of GNP film due to pure shear strain, we attach the gauge axis of the film in alignment with the midline of a cylindrical shaft (see **Figure** **2**b). Once a shaft is loaded by a torque, the principal strains are received at an angle of 45° (see Mohr's circle in Figure 1a). Since the force also results in a bending moment, we calculate the position where no bending moments exist. It is common to measure the induced strain due to torque load in a half bridge of conventional V‐shaped SG as shown in Figure 2b. The results in Figure 2c show that the maximum relative resistance change is 0.001 during a measured principal strain with amplitude of 2160 microstrain. The sensitivity for the s‐PRE is below 1.5% of the l‐PRE and below 3% of the t‐PRE. During a shear strain the geometry of a square conductor changes in the form that as one diagonal comes closer, the distance in the other diagonal increases so that resistance in gauge axis is nearly unchanged.

To understand aforementioned behavior, in the following a model‐based approach is chosen to analyze our experimental results.

### 2.2 Modeling and Simulation

**Figure 3:**
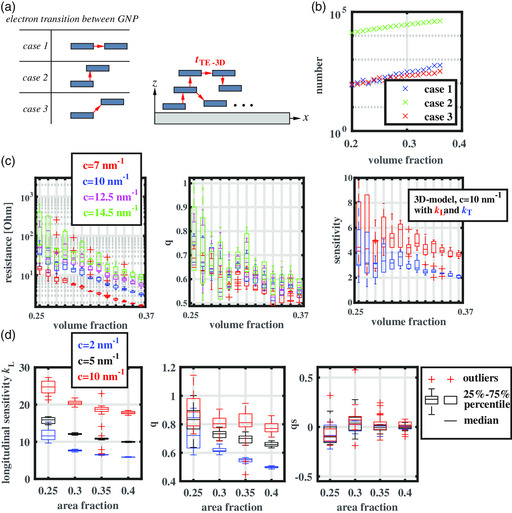
a) Cluster formation and cases of contacts between adjacent GNP. b) Number of the different cases within a percolated 3D model. c) Electromechanical results of the 3D model and d) 2D model respectively.

We assume that in a conductive network the electron transitions between adjacent GNP, which are arranged parallel to the substrate's surface and occupied by surfactants, describe a tunneling gap “in‐plane” (case 1) and “out‐of‐plane” direction (case 2), as shown in **Figure** **3**a. In a further case, the tunneling gap (characterized by the vector t_TE‐3D_) is diagonally arranged (case 3). First, we schematically analyze the electromechanical behavior in a 3D architecture and simulate a monodisperse network of circular‐shaped GNP with a radius of 200 nm and a thickness of 1 nm inside a representative volume size (cubic dimension: length = 5000 nm, width = 3000 nm, and height = 300 nm). While the GNP are randomly set inside the volume element and the percolation of the network is given, aforementioned three cases of contacts between adjacent GNP exist, where the case 2 is the predominant case due to the high aspect ratio of the sheets (see Figure 3b). In that case, the network's behavior is referred to changes of the overlapping area of adjacent GNP due to the examined deformation in‐plane direction (*x*‐ or *y*‐direction). Since the number of contacts representing cases 1 and 3 is two orders lower, we do not consider these cases further. Moreover, we assume that while the substrate is subjected to longitudinal or transverse strain, respectively, the GNP undergo a rigid body motion related to the in‐plane deformation field of the substrate due to slippage effects.^[^ ^3^ ^]^ The network is investigated for a principal strain of 2000 microstrain. The adjacent resistance between the *i*th and *j*th GNP is calculated according to Equation (1)
(1)

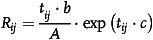

where *t* is the tunneling distance between the GNP (out‐of‐plane distance in *z*‐direction), *b* and *c* are parameters derived from Simmons^[^ ^23^ ^]^ and contain physical constants and the potential barrier (see Section 4, Supporting Information). The contact area *A* represents the overlapped area for each connected pair of GNP, when we consider a cut‐off distance of 1 nm in *z*‐direction. The intrinsic resistance of GNP is neglected and does not have any effects to electromechanical behavior. The network is a circuit of nodes, where each GNP represents a node. In accordance to Kirchhoff's circuit law, the node analysis is given by Equation (2)

(2) $$(\begin{array}{cccc} G_{11} & -G_{12} & \ldots & -G_{1\;\;n-1}\\ -G_{21} & G_{22} & \ldots & -G_{2\;\;n-1}\\ \ldots & \ldots & \ldots & \ldots \\ -G_{n-1\;\;1} & -G_{n-1\;\;2} & \ldots & G_{n-1\;\;n-1} \end{array})(\begin{array}{c} \varphi_{1}\\ \varphi_{2}\\ \vdots \\ \varphi_{n-1} \end{array})=(\begin{array}{c} I_{1}\\ I_{2}\\ \vdots \\ I_{n-1} \end{array}),G_{ij}=G_{ji}\forall i,j\;I_{2},I_{3},\ldots ,I_{n-1}=0,\;i,j=1,2,\ldots ,n-1$$

where *G*_*ij*_ is the admittance between the *i*th and *j*th node, *φ*_*i*_ are the node potentials and *I*_1_ is the current input into the first node of the network. We define the potential at the last node with *φ*_*n*_ = 0. Inside the electrodes all GNP are connected with negligible resistances. Consequently, the contact resistance of the electrodes is not considered in this investigation since it does not represent the network behavior and can be eliminated by a four‐point probe measurement system in practice. The electrodes are considered over the entire width and height of the representative element. Since the potential between the nodes are calculated, the current between the nodes is determined by Ohm's law (see Section 4, Supporting Information). The results in Figure 3c indicate a higher resistance by increasing of the value *c* and decreasing of the volume fraction for an undeformed state, which is obvious and can also be seen in our simulation results. Moreover we examine a higher longitudinal sensitivity than transverse sensitivity considering the same initial state of the GNP network, which is represented by the value of *q* (=*k*_T_/k_L_) below 1 in Figure 3c. Whiskers are drawn from the ends of the interquartile ranges to the furthest adjacent value from the top or bottom of each box (interquartile range under the assumption of normal distributed data). In this context, outliers are values more than 1.5 times outside of the interquartile range. Since the electromechanical behavior is referred to overlapping changes of contact areas, we determine an overall gauge factor of below 10 within the simulated 3D model. The electromechanical sensitivities to a longitudinal (red lines) or transverse strain (blue lines) of 2000 microstrain are exemplary shown for *c* = 10 nm^−1^. Since in experiment the electromechanical response leads to higher sensitivities, we also examine a network attributed to tunneling junction in‐plane direction. As shown by Li et al.,^[^ ^24^ ^]^ the order of magnitude of resistivity change due to change of tunneling distances is significantly higher so that we reduce the piezoresistive behavior by a description in tunneling model space in two dimension, which is relevant for the considered plane deformation field. Therefore, the following simplifying assumption does not describe the particle size distribution or geometry of GNP inside a real architecture, but may offer knowledge about electromechanical behavior once the network is mainly determined by in‐plane tunneling distance changes (see cases 1 and 3). Consequently, the change of intrinsic resistance of GNP or the overlapped areas between GNP are neglected. As aforementioned, the model is limited to tunneling junctions between GNP, which are randomly distributed within the network. The GNPs are reduced to impermeable hardcores in tunneling model space, which are circular in shape for simplicity. We are interested in relative changes of the different strain sensitivities considering the same network, while it undergoes different strains, respectively. We assume that the smallest geometric dimension of GNP, where the tunneling effect does not occur, is 1 nm which correspond to the hardcore diameter. The hardcores are randomly distributed inside a rectangular element. Two cores are considered connected if the distance between their circles is less than the cut‐off distance of 2 nm. Greater distances have negligible influence to overall resistances. As a representation for that criterion, we consider a softshell with a diameter of 3 nm for each core. Different area fractions of occupied hardcores in a randomly distributed field illustrate the main relation for the electromechanical responses *q* (=*k*_T_/*k*_L_) and *qs* (=*k*_S_/*k*_L_) with varying parameter *c* (see box plots in Figure 3d). Relative effects of area size can be neglected by a sufficiently large representative area size of 350 nm × 350 nm of tunneling model space (at least 9500 tunneling junctions). Each simulation is repeated 12 times to ensure statistical validation for the obtained results in the analyzed 3D or 2D models.

The outcome of the simulations confirms that the lower the occupied area fraction, the greater the median of *q* and the greater results spread. The reduction of area fraction goes hand in hand with the reduction in the number of tunnel junctions, so that greater importance is assigned to the single tunnel junctions. As a result, the deviation increases in a randomly distributed network, too. The median also changes, since the randomly distributed hardcores show a different density in the undeformed midline, so that the lowest transverse sensitivity for the highest density of tunneling junctions is examined. Further guideline is provided by variation of value *c*, which is above all a function of the potential barrier. On the one hand, an increase of *c* is associated with higher sensitivities for both, the l‐PRE and t‐PRE. On the other hand, the midline effect is reduced with increasing *c* so that the transverse sensitivity becomes higher, considering the same occupied area fraction. In contrast to that the shear strain sensitivity becomes negligible as already seen in experimental studies in Figure 2c. The relation between the shear strain and the relative resistance change is described by a proportional factor (shear sensitivity). In conclusion, we can say that the simulation results are in agreement with the preliminary experimental studies and provide additional guidelines for the PRE in context of multiaxial strain sensing.

### 2.3 Linear Model of Piezoresistive Behavior

The electromechanical behavior of our spray deposited GNP films typically exhibits linear characteristics as shown in Section 3, Supporting Information, which is already determined in previous works^[^ ^22^ ^]^ and also shown for other carbon allotropes.^[^ ^25^ ^]^ To tackle the behavior with different strain sensitivities in context of an unknown strain field, a tensorial description of the piezoresistive effect is needed.^[^ ^13^ ^]^ Considering the outcome of our preliminary experimental investigations as well as the simulation results, we propose that the electromechanical sensitivities related to different strain components for a gauge line are expressed by Equation (3).

(3) $$r_{i}=\frac{\Delta R_{i}}{R}=k_{L}\cdot \varepsilon_{\text{Li}}+k_{T}\cdot \varepsilon_{\text{Ti}}+k_{S}\cdot \varepsilon_{ij}$$

where *ε*_L_ and *ε*_T_ are strain components in longitudinal and transverse direction and *ε*_*ij*_ is the shear strain for the *i*th gauge line with corresponding piezoresistive sensitivities *k*_L_, *k*_T_, and *k*_S_. The shear dependency of the resistance may be nonlinear. Nevertheless, the effect of the shear should also be taken into account within the linear modeling of the PRE, because the error is seen as negligible due to the relatively low s‐PRE. Moreover, since it can be assumed that the sensitivities of the measuring directions within a sample or when using several samples are subjected to deviations, the results are prioritized using at least three measuring directions by introducing the third unknown of *k*_S_. Bridgeman also introduced factors which denote the proportionality between resistance change and corresponding strain components for polycrystalline metals.^[^ ^10^ ^]^ Smith describes elastoresistive coefficients for semiconducting materials, which characterize the relation between the piezoresistive coefficients and the surface strain components in a similar way.^[^ ^17^ ^]^ Our approach neglects the strain effect within the GNP film perpendicular to the substrate's surface, since we observe a laminar arrangement of densely packed GNP sheets with Van der Waals distances between the particles so that the network deformation is mostly dominated by in‐plane slippage effects as a result of the transmitted surface strain. Hence, we assume that the interparticle distances normal to the substrate's surface remain unchanged.

## 3 Multiaxial Strain Sensing with GNP Film

### 3.1 Geometry of GNP Film

**Figure 4:**
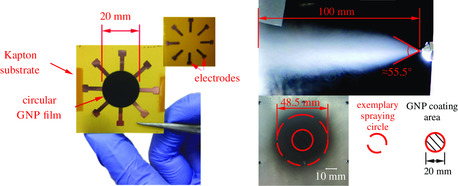
(Left) Circular GNP film with 8 electrodes (here) or 16 electrodes; (right) spraying cone and corresponding omnidirectional spraying area.

In practice all strain components mostly occur simultaneously so that multiple gauge axes are required for a holistic quantification of the piezoresistive behavior. To exclude the influence of differences in PRE of different GNP films, a single GNP film in circular shape is chosen (see **Figure** **4**), which beside symmetry, offers also manufacturing advantages. The two‐substance nozzle of the used spraying device, which works automatically, guarantees a continuous spraying flow. Neither the nozzle nor the stage are moved during the spraying process so that the position is kept fixed to minimize perturbation and to achieve an almost omnidirectional spraying pattern with nearly the same resistance in different directions. Since the particles are randomly deposited, isotropic piezoresistive behavior is assumed. The number and position of the electrodes are taken into account according to the methodology. To apply the measurement geometry to any structures, a carrier substrate in the form of a flexible Kapton film is to be used, to which the electrodes are also applied. A detailed description of the fabrication of our strain sensors is explained in Section 1, Supporting Information. A circular shaped device has recently been published for characterization of CNT with different orientation of electrode pairs attached to a composite substrate, clamped to the tensile machine.^[^ ^25^ ^]^ The aim of that work was only to investigate the PRE for different tensile directions. It was not considered that the underlying substrate is only two times higher in its diameter than the attached sensor, which may result in strain gradients.

### 3.2 Numerical Methodology for Strain Reconstruction Using SD‐EIT

The theory of electrical impedance tomography (EIT) is a topic on its own and shall be considered elsewhere.^[^ ^26^ ^]^ The piezoresistive model in Equation (3) describes a dependency of the change in resistance on the current flow direction itself. This represents an anisotropic problem in the context of EIT that has no unique solution.^[^ ^27^ ^]^ A novel approach to formulate the EIT problem is offered by considering the strain components of a strain rosette as model parameters ***m***, which are a class of isotropic model parameters so that we can use the standard EIT. According to Adler and Guardo,^[^ ^28^ ^]^ the nonlinear EIT problem can be linearized for small changes in the conductivities (see Equation (4)), which is the case for the underlying strain up to around 2000 microstrain. Using the differential EIT also offers additional advantages so that uncertainties (e.g., modelling error) become unimportant.^[^ ^26^ ^]^ The combination of both leads to a novel approach, called SD‐EIT. At the beginning an initial estimate of the contact impedances and homogenous conductivity of the GNP films are done based on the reference data ***v***_0_. The differential data set is determined between the unstrained state ***v***_0_ and strained state ***v***_1_. The Jacobian matrix ***J*** is determined by the perturbation of model parameters. The PRE is adapted elementwise to the finite element model for known electromechanical sensitivities. The task is to find the solution for Δ ***m*** that minimizes the objective function in SD‐EIT, and is given by

(4) $$\underset{\Delta m}{\arg\min}\{{\left\|J\Delta m-\left(v_{1}-v_{0}\right)\right\|}_{2}^{2}\}$$

Using the Levenberg–Marquardt method, the solution of the reconstructed strain state is given by

(5) $$\begin{array}{c} \Delta m={\left(J^{T}J+\lambda \cdot diag(J^{T}J)\right)}^{-1}J^{T}(v_{1}-v_{0})\\ \text{with}\;\Delta m=[\begin{array}{c} \varepsilon_{a,1}-\varepsilon_{a,0}\\ \varepsilon_{b,1}-\varepsilon_{b,0}\\ \varepsilon_{c,1}-\varepsilon_{c,0} \end{array}] \end{array}$$

Once the sensitivities are not known, an additional objective function with a different problem formulation, where now the model parameters $\tilde{m}$ represent the electromechanical sensitivities, is needed. The reference data ***v***_0,FEM_ is calculated by additionally considering the distorted shape of the finite element model due to adapted strain during calibration. The Jacobian matrix $\tilde{J}$ is solely determined through perturbation of piezoresistive sensitivities instead. The task is to find the solution for $\tilde{m}$ that minimizes the objective function, and is given by

(6) $$\underset{\tilde{m}}{\arg\min}\{{\left\|\tilde{J}\tilde{m}-\left(v_{1}-v_{0,FEM}\right)\right\|}_{2}^{2}\}$$

where ***v***_1_ is the data vector for strained GNP film. The solution is found using the Moore–Penrose inverse, given by Equation (7) (using the function pinv in Matlab)

(7) $$\begin{array}{c} \tilde{m}={\left({\tilde{J}}^{T}\tilde{J}\right)}^{-1}{\tilde{J}}^{T}(v_{1}-v_{0,FEM})\\ \text{with}\;\tilde{m}=[\begin{array}{c} k_{L}\\ k_{T}\\ k_{S} \end{array}] \end{array}$$

In this article, we apply the SD‐EIT for a Sheffield protocol^[^ ^26^ ^]^ with 16 electrodes, which offers the advantage of four point measurement.

### 3.3 Analytical Model for Strain Reconstruction (SR‐AM)

**Figure 5:**
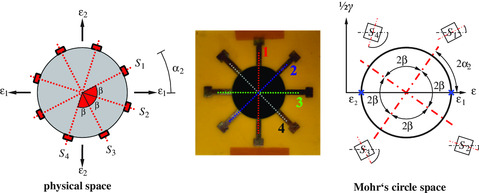
GNP film which is exemplary subjected to the principal strains *ε*_1_ and *ε*_2_ (physical space) and where its strain components in different gauge axes are determined in Mohr's circle space.

A further analytical approach is derived from the numerical model with the elementwise implemented model of PRE (see Section 2, Supporting Information). A linear electromechanical relation between the strain components and two oppositely driven electrodes (defined as gauge axis or gauge line) is also observed for a circle resistor so that we derive an additional analytical model for strain reconstruction (SR‐AM). It must be observed that in a circle the corresponding current paths are not straight and crosstalk between electrodes exist. In this case, the empirical PRE model (see Equation (3)) with the strain components cannot be referred to a straight direction of current path, as it is assumed for a rectangular shaped single resistor or elements of the finite element model as discussed in Section 3.2. To express the strain components in context of the circle resistor, multiple gauge lines 45° apart are introduced as shown in **Figure** **5**. The sensitivities *k*_L,Z_, *k*_T,Z_, and *k*_S,Z_ represent the electromechanical behavior referred to the gauge axes within the circular GNP film and differ from electromechanical sensitivities, which are related to single resistors with straight lines (therefore, we add the subscript Z). Once the sensitivities are determined, at least three gauge lines to determine three unknown strain components are needed. By implementing the fourth gauge line, a symmetrical sensor structure is realized. In addition, we develop a simple procedure to determine sensor faults. An assumption of a quasi‐isotropic film with randomly oriented GNP is accompanied by integration of all gauge axes within the circle.

The strain components at a specific point of any relative angular orientation of gauge axis indicate the Mohr's strain circle for an isotropic substrate so that we formulate the relation between the relative resistances changes *r*_i_ and uniform sensitivities in three gauge axes, as given by Equation (8)

(8) $$\left[\begin{array}{c} r_{1}\\ r_{2}\\ r_{3} \end{array}]=[\begin{array}{ccc} \varepsilon_{L1} & \varepsilon_{L3} & 2\varepsilon_{L2}-\varepsilon_{L1}-\varepsilon_{L3}\\ \varepsilon_{L2} & \varepsilon_{L1}+\varepsilon_{L3}-\varepsilon_{L2} & \varepsilon_{L3}-\varepsilon_{L1}\\ \varepsilon_{L3} & \varepsilon_{L1} & \varepsilon_{L1}+\varepsilon_{L3}-2\varepsilon_{L2} \end{array}][\begin{array}{c} k_{L,Z}\\ k_{T,Z}\\ k_{S,Z} \end{array}\right]$$

where *ε*_Li_ represents the longitudinal strain for the *i*th gauge axis. A complete derivation of Equation (8) is shown in Section 2, Supporting Information. Once the sensitivities are determined (e.g., from a previous calibration) and relative resistances in three axes 45° apart are measured, the corresponding longitudinal strains in gauge axes can be calculated, as given in Equation (9)

(9) $$\left[\begin{array}{c} \varepsilon_{L1}\\ \varepsilon_{L2}\\ \varepsilon_{L3} \end{array}]={\left[\begin{array}{ccc} k_{L,Z}-k_{S,Z} & 2k_{S,Z} & k_{T,Z}-k_{S,Z}\\ k_{T,Z}-k_{S,Z} & k_{L,Z}-k_{T,Z} & k_{T,Z}+k_{S,Z}\\ k_{T,Z}+k_{S,Z} & -2k_{S,Z} & k_{L,Z}+k_{S,Z} \end{array}\right]}^{-1}[\begin{array}{c} r_{1}\\ r_{2}\\ r_{3} \end{array}\right]$$

In case of a fourth gauge axis the linear system of equations is overdetermined, as given in Equation (10)

(10) $$\left[\begin{array}{c} r_{1}\\ r_{2}\\ r_{3}\\ r_{4} \end{array}]=[\begin{array}{ccc} \varepsilon_{L1} & \varepsilon_{L3} & 2\varepsilon_{L2}-\varepsilon_{L1}-\varepsilon_{L3}\\ \varepsilon_{L2} & \varepsilon_{L1}+\varepsilon_{L3}-\varepsilon_{L2} & \varepsilon_{L3}-\varepsilon_{L1}\\ \varepsilon_{L3} & \varepsilon_{L1} & \varepsilon_{L1}+\varepsilon_{L3}-2\varepsilon_{L2}\\ \varepsilon_{L1}+\varepsilon_{L3}-\varepsilon_{L2} & \varepsilon_{L2} & \varepsilon_{L1}-\varepsilon_{L3} \end{array}][\begin{array}{c} k_{L,Z}\\ k_{T,Z}\\ k_{S,Z} \end{array}\right]$$

so that the best solution for electromechanical sensitivities can be found by means of least squares^[^ ^29^ ^]^ (using the function pinv in Matlab). Once the sensitivities are also determined and relative resistances in four axes 45° apart are measured, the longitudinal strains in three gauge axes can be calculated by the pseudoinverse of the sensitivity matrix, given in Equation (11)

(11) $$\left[\begin{array}{c} r_{1}\\ r_{2}\\ r_{3}\\ r_{4} \end{array}]=[\begin{array}{ccc} k_{L,Z}-k_{S,Z} & 2k_{S,Z} & k_{T,Z}-k_{S,Z}\\ k_{T,Z}-k_{S,Z} & k_{L,Z}-k_{T,Z} & k_{T,Z}+k_{S,Z}\\ k_{T,Z}+k_{S,Z} & -2k_{S,Z} & k_{L,Z}+k_{S,Z}\\ k_{L,Z}+k_{S,Z} & k_{T,Z}-k_{L,Z} & k_{L,Z}-k_{S,Z} \end{array}][\begin{array}{c} \varepsilon_{L1}\\ \varepsilon_{L2}\\ \varepsilon_{L3} \end{array}\right]$$

The condition number of the sensitivity matrix is ≈1.4 so that results will be stable. The derived equations ((10) and (11)) can also be applied to a rosette of four single GNP films, which are arranged in the same way as shown in the circular shaped resistor. However, the single circle resistor with multiple electrodes has the advantage to verify the results based on two different methods, which are presented in this article (SD‐EIT and SR‐AM). EIT can also be used to obtain conductivity changes through damages on GNP film, which is not possible by using single resistors. Moreover, the proposed methods for principal strain reconstruction consider GNP films with identical electromechanical sensitivities in different gauge lines which may be more difficult to achieve in case of multiple films due to fabrication uncertainties. In the case of a single circular shaped GNP film, which is fabricated by a nozzle with omnidirectional spraying pattern, we almost achieve the same resistance across the different gauge lines.

### 3.4 Proof of Concept

#### 3.4.1 SD‐EIT

**Figure 6:**
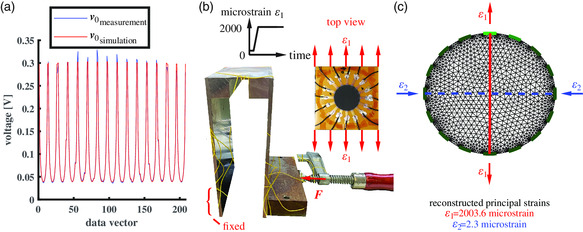
a) Measured and simulated data vector ***v***_0_ with 208 measurement points (Sheffield protocol); b) experimental measurement setup for SD‐EIT (see also Figure 1); c) reconstructed strain (red line corresponds to principal strain direction *ε*_1_, dashed line corresponds to principal strain direction *ε*_2_).

In the SD‐EIT method, recording of the data vector ***v***_0_ begins and ends with a trigger signal and is related to the Sheffield protocol of 208 data points. The voltage signal of an electrode pair is measured for 0.5 s at a frequency of 200 Hz. The measured experimental data points and the initial estimation for a finite element model with a homogenous conductivity and 16 line electrodes are plotted in **Figure** **6**a. In our case, the mean value of the absolute deviation over all measured voltage values is 6.15% ± 3.6%. Since the SD‐EIT is based on a one‐step differential approach, the corresponding modeling errors vanish. In a first step, the piezoresistive sensitivities are determined according to Equations (6) and (7). The examined values are *k*_L_ = 5.53, *k*_T_ = 3.12, and *k*_S_ = 0.1, which represent a GNP film with low sensitivities. Nevertheless the results of the preliminary investigation are further confirmed (*k*_L_ > *k*_T_ >> *k*_S_). To reconstruct these values we use a numerical model with 3474 elements. The number of elements is considered to be sufficiently large so that numerical errors can be neglected. In a second step, the principal strain components *ε*_1_ and *ε*_2_ with the principal angle are determined by the solution of the model parameters *ε*_a_, *ε*_b_ and *ε*_c_ according to Equation (5). To avoid inverse crime, the numerical model to determine the strain components differs from the numerical model to calculate the electromechanical sensitivities. In the SD‐EIT, we reconstruct a principal strain components with *ε*_1_ = 2003.6 microstrain and *ε*_2_ = −2.3 microstrain at a principal strain angle of 0.2° to the vertical (see Figure 6c). The result from SD‐EIT of the principal strains are additionally verified by metallic SG, which is attached at the center of the uniaxial strained device in Figure 6b and represents a strain of 2005 microstrain. Considering the comparatively lower sensitivities of the sample, which can make the determination of strain even more difficult due to possible fluctuations in measured values, the results show a high accuracy of 99.7% so that the plane strain determination of the SD‐EIT is successfully given within the experiment. The small deviations to signal of SG can be explained since the GNP sample may not represent an ideal geometry or alignment errors may arise during bonding.

#### 3.4.2 SR‐AM

**Figure 7:**
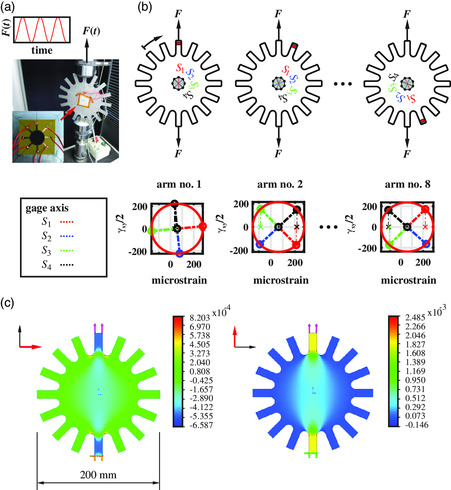
a) Photo of the sensor attached to the steel plate specimen. b) Orientation of the gauge axes *S*_1_ to *S*_4_ during multidirectional tensile testing with its corresponding strain components in Mohr's circle space (lower figures). c) Finite element analysis of the specimen with the corresponding strain in transverse direction (left) and longitudinal direction (right).

A new measurement setup which on the one hand allows quantifying our sensor in a nearly homogeneous strain field and on the other hand enables different principal strain directions is used in the context of SR‐AM. The sensor is attached at the center, where the strain gradients in longitudinal and transverse directions become insignificant. The finite element analysis (see **Figure** **7**c) yields results for the principal strain *ε*_1_ in a value range of 319.07 ± 2.26 microstrain with the ratio *ε*_2_/*ε*_1_ = 0.593 ± 0.001 in the measurement area. Since the maximum deviation from the mean *ε*_1_ is below 1%, a homogeneous strain field is assumed for the position, where the GNP is attached. In our experiment the specimen is clamped and strained at a frequency of 1 Hz in a hydropulse machine (see Figure 7a). To characterize the PRE in multiple principal strain directions the specimen is turned counterclockwise as shown in Figure 7b. The resulting Mohr's circle and the strain components in the different gauge axes (shown in Figure 7b) are determined by three additional metallic strain gauges, which are attached to the back and aligned to the gauge axes *S*_1_, *S*_2_, and *S*_4_ of our sensor.

**Figure 8:**
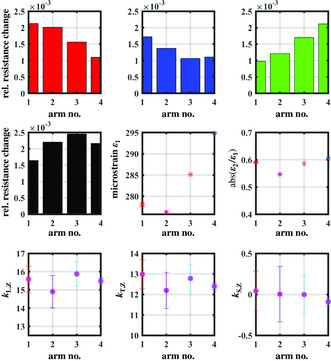
(Top) Relative resistance changes of *S*_1_ to *S*_4_ during multidirectional tensile testing. (mid‐row) absolute and relative values of principal strains while the tensile axis is turned counterclockwise in steps of 22.5°. (bottom) Longitudinal (left), transverse (center) and shear sensitivity for SR‐AM (right).

**Figure 9:**
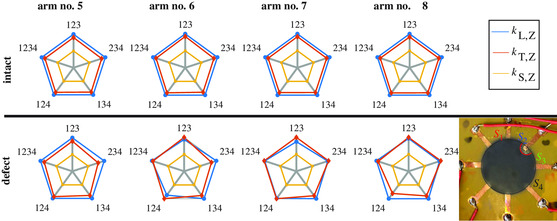
(Top) Examined sensitivities for different constellation of considered gauge axes for a healthy GNP film. (Bottom) Examined sensitivities for different constellation of considered gauge axes for a faulty GNP film at transition to electrode in gauge axis *S*_2_.

We calculate the different sensitivities according to Equation (10), depending on the number of gauge axes which are considered for sensitivity analysis. Since the strain components, which are described in Mohr's circle space, are repeated after 90°, it is sufficient to measure arm nos. 1 to 4 (quarter circle of the specimen). In **Figure** **8** the relative resistance changes of the four gauges are shown as a function of strained arm number. Each value represents the median of at least 30 measurements. As a first estimate, since all axes are more sensitive to longitudinal strain, we can conclude that when pairs of rectangular aligned gauge axes (*S*_1_–*S*_3_ or *S*_2_–*S*_4_) are in line to the principal axes, the ratio between the minimal and maximal relative resistance change is the highest. It is interesting to notice that although we are using four different configurations of considered gauge axes (as seen also in **Figure** **9**), the sensitivities using least squares remain in the same range (see Figure 8a). While the longitudinal sensitivities for SR‐AM are in a range of 15.46 ± 0.41, the shear sensitivities vary around zero. The ratio of transverse sensitivity to longitudinal sensitivity is 0.81 ± 0.01. The principal strain directions can be calculated for the sensitivity matrix (see Equation (11)) according to the known rosette theory (see Section 5). Using the overall mean for corresponding electromechanical sensitivities within the sensitivity matrix, we determine principal angles of −1.6°, 23.4°, 48°, and 67.1° during loading of the four arms. The results come very close to theoretical principal angles of 0°, 22.5°, 45°, and 67.5° (see Figure 7). The deviation of the results may be explained by small inhomogeneity in the piezoresistive behavior in different direction. Despite that the substrate shows a slight stiffness deviation in different directions (see Figure 8 mid‐row) which may also affect the calculation of electromechanical sensitivities. Finally, small alignment errors may arise during clamping of specimen.

Since our approach provides four gauge axes, a strategy for sensor fault detection shall finally be validated using the overdetermined linear equation system (see Equation (10)). As long as the sensor is intact, we observe similar strain sensitivities using the signal of different gauges axes within the sensor, which can be seen in the spider plot in Figure 9. Afterward we introduce a faulty sensor removing the GNP film partially at the transition to the electrode for gauge axis *S*_2_. The reconstructed results in 134 (corresponding to sensitivities determination using gauge axes *S*_1_, *S*_3_, and *S*_4_) solely indicate the similar behavior as seen in the healthy state of GNP film. All other piezoresistive sensitivities, where gauge axis *S*_2_ is considered, are influenced by the faulty state. The higher the gauge axis of *S*_2_ is strained in longitudinal direction, the higher the anomalies in sensor's performance are seen. Due to the introduced defect within the GNP film, we examine results where the spider plots are not symmetrical anymore (see Figure 9). We can conclude that we successfully detect faulty GNP with SR‐AM so that this information can be used to further evaluate electromechanical behavior of GNP film and increase sensor's robustness in future, which is also a crucial point in sensor development.

## 4 Conclusion

The problem of multiaxial strain sensing with piezoresistive films of nanomaterials is mostly underestimated or misunderstood and therefore not accurately quantified in scientific papers. To tackle that problem, we investigated the sensitivities with respect to the gauge axis to different well‐defined strain components separately. Our results showed that GNP piezoresistive films are affected in a different way, depending on the direction of strain with respect to the current flow. While the longitudinal strain sensitivity is the highest, the transverse sensitivity exceeds 50% of the longitudinal sensitivity and cannot be neglected. The shear strain sensitivity is the smallest and varies around zero. The piezoresistive effect to different strain component was examined in a 3D model with overlapping adjacent GNP and by a hardcore‐softshell 2D‐model in a simplified tunneling model space. Those findings from simulation help to validate our experimental results and to find an optimized sensor design for our GNP film. We developed an empirical piezoresistive model for GNP film. Finally, we introduced a novel numerical (SD‐EIT) and analytical approach (SR‐AM) to multiaxial strain sensing. The strain reconstruction is successfully proved by a circular shaped GNP film for both methods. The analytical approach SR‐AM was further used to characterize sensor defects, which increase also the robustness of sensor's performance. All these theoretical and experimental investigations may have an important role in the design of piezoresistive strain gauges based on nanomaterials. In future works, the sensor performance can be statistically validated. In addition the SD‐EIT can be extended for model parameters, which enable the reconstruction of spatial strain with gradients within a plane strain field.

## 5 Experimental Section

5.1

5.1.1

##### Materials

Powder of GNP (trade name: Elicarb SP 8073 powder) was purchased from Thomas Swan. The GNP were dissolved in a fixed concentration of 1 mg ml^−1^ (0.1 wt%). Sodium cholate (for samples in preliminary investigations or SR‐AM) or cocamidopropybetaine (samples for SD‐EIT) were used as surfactant.

##### Methods

A material characterization (e.g., Raman spectroscopy) for the spray deposited GNP films is carried out in Section 1, Supporting Information. The Raman spectra of GNP films were measured on polyimide substrate with a WITec alpha 300R system and a 532 nm laser for excitation at room temperature.

##### Suspension Preparation

Suspension containing GNP concentration of 1 mg ml^−1^ were mixed with surfactant in deionized water. To achieve a higher degree of absorption on the GNP surface, the preparation of suspension was stirred and sonicated (ultrasonicator from Hielscher GmbH, amplitude of 60%, cycle 0.5 Hz, tip diameter 0.7 mm) for the duration of 30 min.

##### Device Fabrication

A flexible polyimide foil with a thickness of 25 μm (trade name: Pyralux HT8515) which includes a double‐sided copper‐clad laminate with a thickness of 18 μm was acquired from Dupont. The copper surface was coated with a nitrate cellulose lacquer using a shadow mask for selective removal. The free surfaces of the copper layers were etched in 40% concentrated FeCl_3_‐solution. The substrate was washed with water, complemented by removing CL with acetone. By this way, the electrodes were fabricated and GNP film was spray coated in 5 × 5 mm rectangular shape. The total spraying amount was fixed to 20 ml for the fabrication of a batch with four GNP films. The deposition was done by the in‐house‐designed spraying system.^[^ ^22^ ^]^ The substrate was attached in a fixed distance of 100 mm to the two substances nozzle. The spraying system operated at a fixed air flow pressure of 1.5 bar and used the Venturi principle. The total spraying amount for the fabrication of the circle was fixed to 40 ml. While spraying of dispersion, the GNP films were allowed to dry onto a heated substrate at 100 °C, followed by thermal annealing in an oven at temperature of 200 °C for 30 min to ensure complete evaporation of the aqueous component. Cross‐sensitivities were reduced by an additional encapsulation layer.

##### Electromechanical Testing

To achieve a uniform strain transfer to polyimide substrate (Young's modulus of 2800 MPa), the specimens were attached to aluminum (uniaxial strain device in Figure 1) or to steel (shaft in Figure 2 or multiaxial strain device in Figure 7) base material using a very thin adhesive layer of cyanoacrylate adhesive (trade name: Z70 from Hottigner Baldwin Messtechnik). The specimen for experimental validation of SR‐AM is mounted in the Schenck hydropulse system PEZ 4125. The electromechanical response due to strained GNP film was evaluated in a Wheatstone bridge circuit (WBC) in SR‐AM. A change in resistance of GNP film disrupts the initial balance of the resistances $\frac{R_{1}}{R_{2}}=\frac{R_{GNP}}{R_{4}}=1$ for the WBC, so that bridge voltage is measured (measurement device: Quantum MX 410B from Hottiger Baldwin Messtechnik). During measurement the bridge supply voltage of 20 V was externally applied with power supply NSP‐3630 from Manson. The reference strains for validation during our experiments were measured through metallic strain gauges with a gauge factor of 2 purchased from Hottigner Baldwin Messtechnik.

## Conflict of Interest

The authors declare no conflict of interest.

## Data Availability Statement

Data available on request from the authors.

## Supporting information

Supplementary Material

## References

[1] a) J. Li , F. Ye , S. Vaziri , M. Muhammed , M. C. Lemme , M. Östling , Adv. Mater. (Deerfield Beach, FL) 2013, 25, 3985; 10.1002/adma.201300361 23728928

[2] a) L. B. Modesto-López , M. Miettinen , J. Riikonen , T. Torvela , C. Pfüller , V.-P. Lehto , A. Lähde , J. Jokiniemi , Aerosol Sci. Technol. 2015, 49, 45;

[3] M. Hempel , D. Nezich , J. Kong , M. Hofmann , Nano Lett. 2012, 12, 5714. 23045955 10.1021/nl302959a

[4] M. Tian , Y. Huang , W. Wang , R. Li , P. Liu , C. Liu , Y. Zhang , J. Mater. Res. 2014, 29, 1288.

[5] A. D. Smith , K. Elgammal , F. Niklaus , A. Delin , A. C. Fischer , S. Vaziri , F. Forsberg , M. Råsander , H. Hugosson , L. Bergqvist , S. Schröder , S. Kataria , M. Östlinga , M. C. Lemme , Nanoscale 2015, 7, 19099. 26523705 10.1039/c5nr06038a PMC4653760

[6] a) M. Amjadi , K.-U. Kyung , I. Park , M. Sitti , Adv. Funct. Mater. 2016, 26, 1678;

[7] a) X. Li , R. Zhang , W. Yu , K. Wang , J. Wei , D. Wu , A. Cao , Z. Li , Y. Cheng , Q. Zheng R. S. Ruoff , H. Zhu , Sci. Rep. 2012, 2, 870; 23162694 10.1038/srep00870 PMC3499758

[8] B. R. Loyola , V. La Saponara , K. J. Loh , T. M. Briggs , G. O’Bryan , J. L. Skinner , IEEE Sensors J. 2013, 13, 2357.

[9] Y. Zhao , M. Schagerl , S. Gschossmann , C. Kralovec , Struct. Health Monit. 2018, 29, 147592171880596.

[10] P. W. Bridgman , Proc. Am. Acad. Arts Sci. 1925, 60, 423.

[11] Y. Kanda , Sens. Actuators A: Phys. 1991, 28, 83.

[12] J. Chen , B. Liu , H. Zhang , Front. Optoelectron. China 2011, 4, 204.

[13] Y. Zhao , S. Gschossmann , M. Schagerl , P. Gruener , C. Kralovec , Smart Mater. Struct. 2018, 27, 105009.

[14] Z. Chen , T. Ming , M. M. Goulamaly , H. Yao , D. Nezich , M. Hempel , M. Hofmann , J. Kong , Adv. Funct. Mater. 2016, 26, 5061.

[15] J. G. Simmons , J. Appl. Phys. 1963, 34, 1793.

[16] X. Wang , J. Li , H. Song , H. Huang , J. Gou , ACS Appl. Mater. Interfaces 2018, 10, 7371. 29432684 10.1021/acsami.7b17766

[17] C. S. Smith , Phys. Rev. 1954, 94, 42.

[18] W. G. Pfann , R. N. Thurston , J. Appl. Phys. 1961, 32, 2008.

[19] Measurement Group, Inc. , Exp. Techn. 1983, 7, 30.

[20] A. S. Fiorillo , C. D. Critello , S. A. Pullano , Sens. Actuators A: Phys. 2018, 281, 156.

[21] a) Sicherheit und Zuverlässigkeit durch experimentelle Struktur- und Beanspruchungsanalyse, VDI-Verlag., Düsseldorf 2001;

[22] V. Yokaribas , S. Wagner , D. Schneider , P. Friebertshäuser , M. Lemme , C.-P. Fritzen , Sensors 2017, 17, 2937. 29258260 10.3390/s17122937 PMC5751506

[23] J. G. Simmons , J. Appl. Phys. 1963, 34, 1793.

[24] C. Li , E. T. Thostenson , T.-W. Chou , Appl. Phys. Lett. 2007, 91, 223114.

[25] T. Xu , Q. Qiu , S. Lu , K. Ma , X. Wang , Int. J. Distrib. Sensor Netw. 2019, 15, 155014771982968.

[26] D. Holder , Electrical impedance tomography. Methods, history, and applications, Institute of Physics Pub, Bristol, PA 2005.

[27] S. J. Hamilton , M. Lassas , S. Siltanen , Inverse Problems 2014, 30, 75007.

[28] A. Adler , R. Guardo , IEEE Trans. Med. Imaging 1996, 15, 170. 18215899 10.1109/42.491418

[29] W. R. Osgood , R. G. Sturm , Bureau Stand. J. Res. 1933, 15, https://nvlpubs.nist.gov/nistpubs/jres/15/jresv15n6p579_A1b.pdf.

