# Supplementary material for: Novel Methodologies for Multiaxial Strain Measurements with Piezoresistive Films based on Graphene Nanoplatelets
Source: Small Sci. 2021 Oct 24;1(12):2100088. doi: 10.1002/smsc.202100088 (PMC11935909; doi:10.1002/smsc.202100088)
Supplement: Supplementary file 1 — Supplementary Material [file SMSC-1-2100088-s001.pdf]

Supporting Information

Novel Methodologies for Multiaxial Strain Measurements with Piezoresistive Films based on Graphene Nanoplatelets

Volkan Yokaribas, Peter Kraemer, Alexander B. Mende; Jasper Ruhkopf, Max C. Lemme, Claus-Peter Fritzen*

Supplementary Section 1. Fabrication & Material characterization

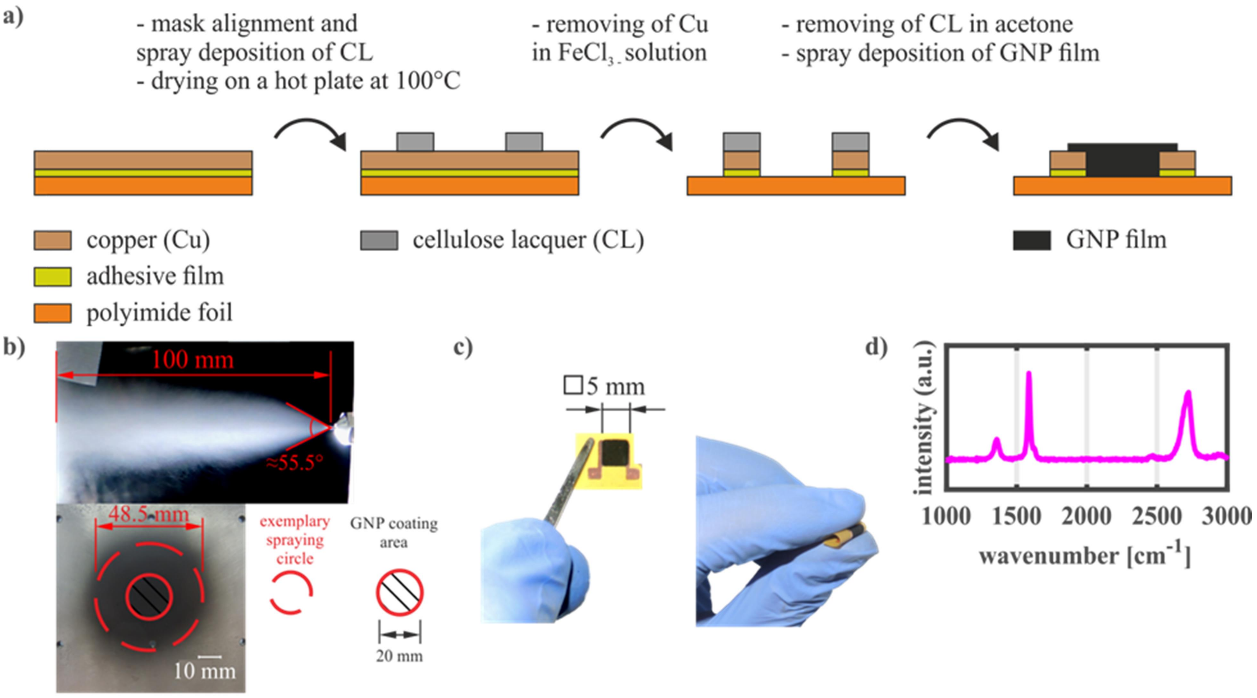

Supplementary Figure S1. (a) Schematically shown fabrication steps of GNP films on Kapton substrate. (b) Spraying cone with an omnidirectional spraying pattern. (c) GNP films used for preliminary investigations. (d) Raman spectrum of GNP film.

Supplementary Section 2.

S2.1 Introduction to SR-AM

Using a two-dimensional Finite Element Model (FEM) (number of elements 3140, 8 electrodes) of a circle with a homogenous conductivity, which shall be considered for a film with random distributed GNP, the potential distribution for oppositely driven electrodes can be exemplary calculated (see figure S2 (left)). With the node potential (Φ_1, Φ_2, Φ_3) and node position in a Cartesian coordinate system (x-y) of the triangular element, the electric field $\boldsymbol{E}$ within an element of the FEM can be derived according to eq. S1.

$$\begin{bmatrix} \Phi_1 \\ \Phi_2 \\ \Phi_3 \end{bmatrix} = \begin{bmatrix} 1 & x_1 & y_1 \\ 1 & x_2 & y_2 \\ 1 & x_3 & y_3 \end{bmatrix} \begin{bmatrix} \Phi_0 \\ E_x \\ E_y \end{bmatrix}. \tag{S1}$$

Using the Ohm’s law, the current flux can be simulated as shown in the figure S2 (right):

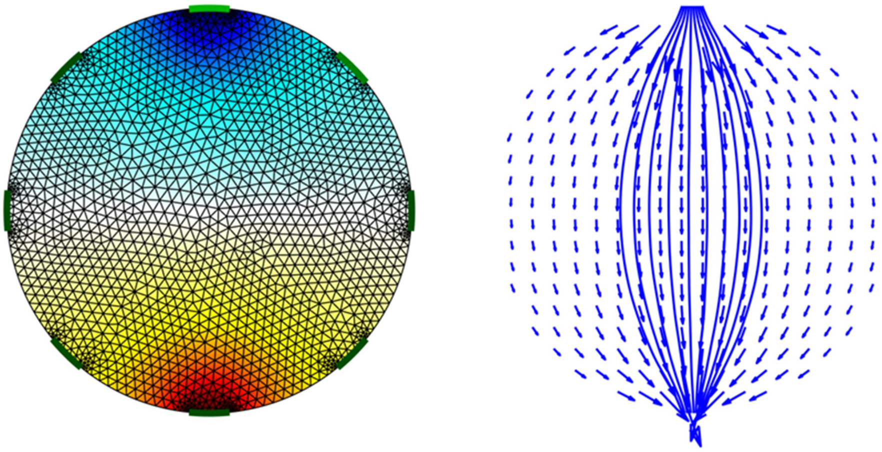

Supplementary Figure S2. (left) node potential distribution and the simulated current path within a circle resistor

The longer the arrow length, the higher is the current strength within the vector field. The extracted area of lines schematically shows the currents paths at the center of the circle model. First of all, it should be noticed that in SR-AM a circle shape is considered where the corresponding current paths are not straight and crosstalk between electrodes exist. In this

case the empirical piezoresistive model (see eq. 3 in the manuscript) with the strain components cannot be referred to a straight direction of current path, as it is assumed for a rectangular shaped single resistor or elements of the finite element model as seen above (see also section 3.2 in the manuscript). Nevertheless, it is further confirmed that a linear electromechanical behavior for resistance measurements of oppositely driven electrodes (defined as gauge line) is also given for the circle resistor. In order to define it more clearly, we simulate the voltage change during uniaxial strain load to the corresponding gauge lines (S1, S2, S3 and S4). To do that we implement the empirical piezoresistive model (according to eq. 3 in the manuscript) **elementwise** to the above shown finite element model of the circle resistor. As aforementioned and as seen in the figure S3, we also determine a linear electromechanical response in this case.

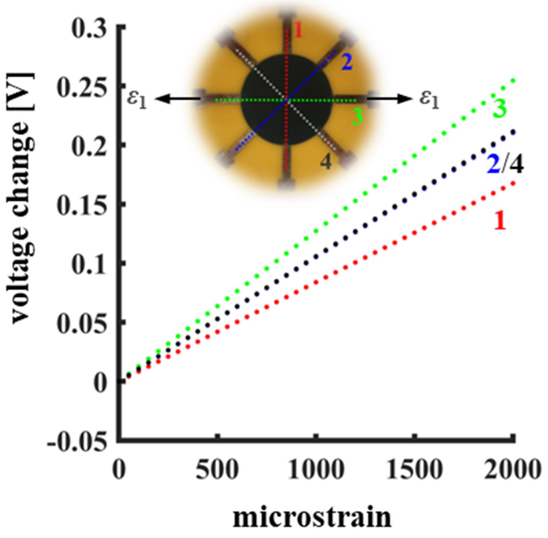

Supplementary Figure S3. Voltage change of the circle resistor

The relation to relative resistance change measured in experiment and the corresponding electromechanical sensitivities within the circle resistor can be calculated according to the given eq. S2.

$$\frac{U_i}{U}\text{-}1=\frac{R_i}{R}\text{-}1=\frac{\Delta R_i}{R}=r_i=k_{L,Z}\cdot\varepsilon_{Li}+k_{T,Z}\cdot\varepsilon_{Ti}+k_{S,Z}\cdot\varepsilon_{ij} \tag{S2}$$

It should be noticed that the electromechanical sensitivities from the circle resistor seen above (therefore we add the subscript Z) differ from electromechanical sensitivities, which are related to single resistors with straight current paths.

S2.2 An extended derivation of the formula for SR-AM

The strain components at a specific point of any relative angular orientation of gauge axis indicate the Mohr's Strain circle. If we take the longitudinal strain and shear strain components of the 1st direction as reference, the other strain components can be expressed with a rotation matrix in Mohr's strain circle space, given by

$$\begin{bmatrix}\varepsilon_{L2}\\ \varepsilon_{24}\end{bmatrix}=\begin{bmatrix}0 & 1\\ -1 & 0\end{bmatrix}\begin{bmatrix}\varepsilon_{L1}\text{-}\varepsilon_m\\ \varepsilon_{13}\end{bmatrix}+\begin{bmatrix}\varepsilon_m\\ 0\end{bmatrix}. \tag{S3}$$

$$\begin{bmatrix}\varepsilon_{L3}\\ \varepsilon_{31}\end{bmatrix}=\begin{bmatrix}-1 & 0\\ 0 & -1\end{bmatrix}\begin{bmatrix}\varepsilon_{L1}\text{-}\varepsilon_m\\ \varepsilon_{13}\end{bmatrix}+\begin{bmatrix}\varepsilon_m\\ 0\end{bmatrix} \tag{S4}$$

$$\begin{bmatrix}\varepsilon_{L4}\\ \varepsilon_{42}\end{bmatrix}=\begin{bmatrix}0 & -1\\ 1 & 0\end{bmatrix}\begin{bmatrix}\varepsilon_{L1}\text{-}\varepsilon_m\\ \varepsilon_{13}\end{bmatrix}+\begin{bmatrix}\varepsilon_m\\ 0\end{bmatrix} \tag{S5}$$

To describe the eq. S3 to S5 with three unknowns, we use the relation for mean strain $\varepsilon_m=\frac{\varepsilon_{L1}+\varepsilon_{L3}}{2}$ and shear strain $\frac{\varepsilon_{13}}{2}=2\varepsilon_{L2}\text{-}\varepsilon_{L1}\text{-}\varepsilon_{L3}$. Furthermore the transverse strain components are replaced by $\varepsilon_{T1}=\varepsilon_{L3}$, $\varepsilon_{T2}=\varepsilon_{L4}$, $\varepsilon_{T3}=\varepsilon_{L1}$ and $\varepsilon_{T4}=\varepsilon_{L2}$ so that the given eq. S6

$$r_i=\frac{\Delta R_i}{R}=k_L\cdot\varepsilon_{Li}+k_T\cdot\varepsilon_{Ti}+k_S\cdot\varepsilon_{ij}. \tag{S6}$$

can be rewritten in the form of eq. S7

$$\begin{bmatrix}r_1\\ r_2\\ r_3\end{bmatrix}=\begin{bmatrix}\varepsilon_{L1} & \varepsilon_{L3} & 2\varepsilon_{L2}-\varepsilon_{L1}-\varepsilon_{L3}\\ \varepsilon_{L2} & \varepsilon_{L1}+\varepsilon_{L3}-\varepsilon_{L2} & \varepsilon_{L3}-\varepsilon_{L1}\\ \varepsilon_{L3} & \varepsilon_{L1} & \varepsilon_{L1}+\varepsilon_{L3}-2\varepsilon_{L2}\end{bmatrix}\begin{bmatrix}k_{L,Z}\\ k_{T,Z}\\ k_{S,Z}\end{bmatrix}. \tag{S7}$$

Supplementary Section 3. Linear electromechanical response of GNP films

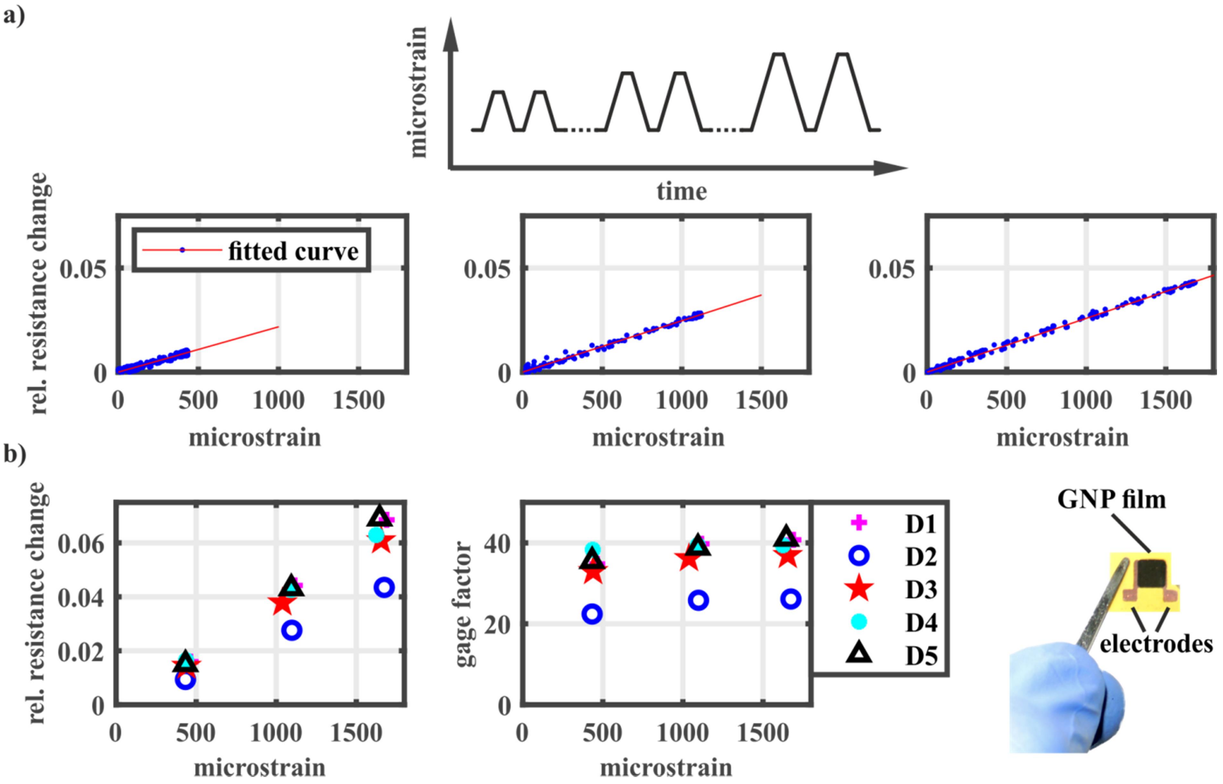

Supplementary Figure S4. (a) Resistance changes as a function of uniaxial, longitudinal strain of a specimen during three increasing load cycles. (b) Relative resistance changes of five specimens and the corresponding gauge factors.

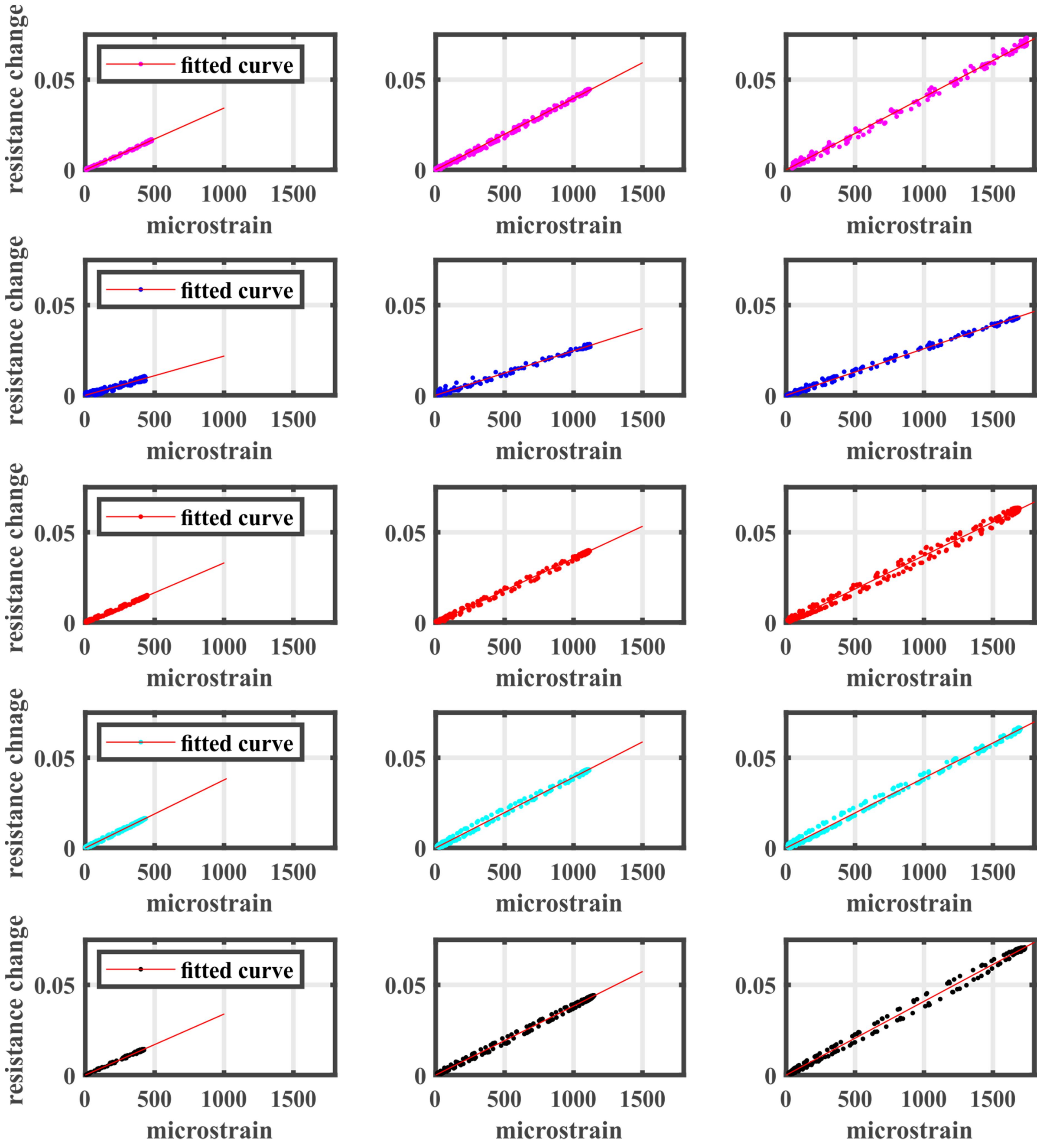

Supplementary Figure S5. Each row represents a specimen shown in figure S4 with estimated gauge factor based on least square optimized fitted linear curves.

Supplementary Section 4. Modeling procedure.

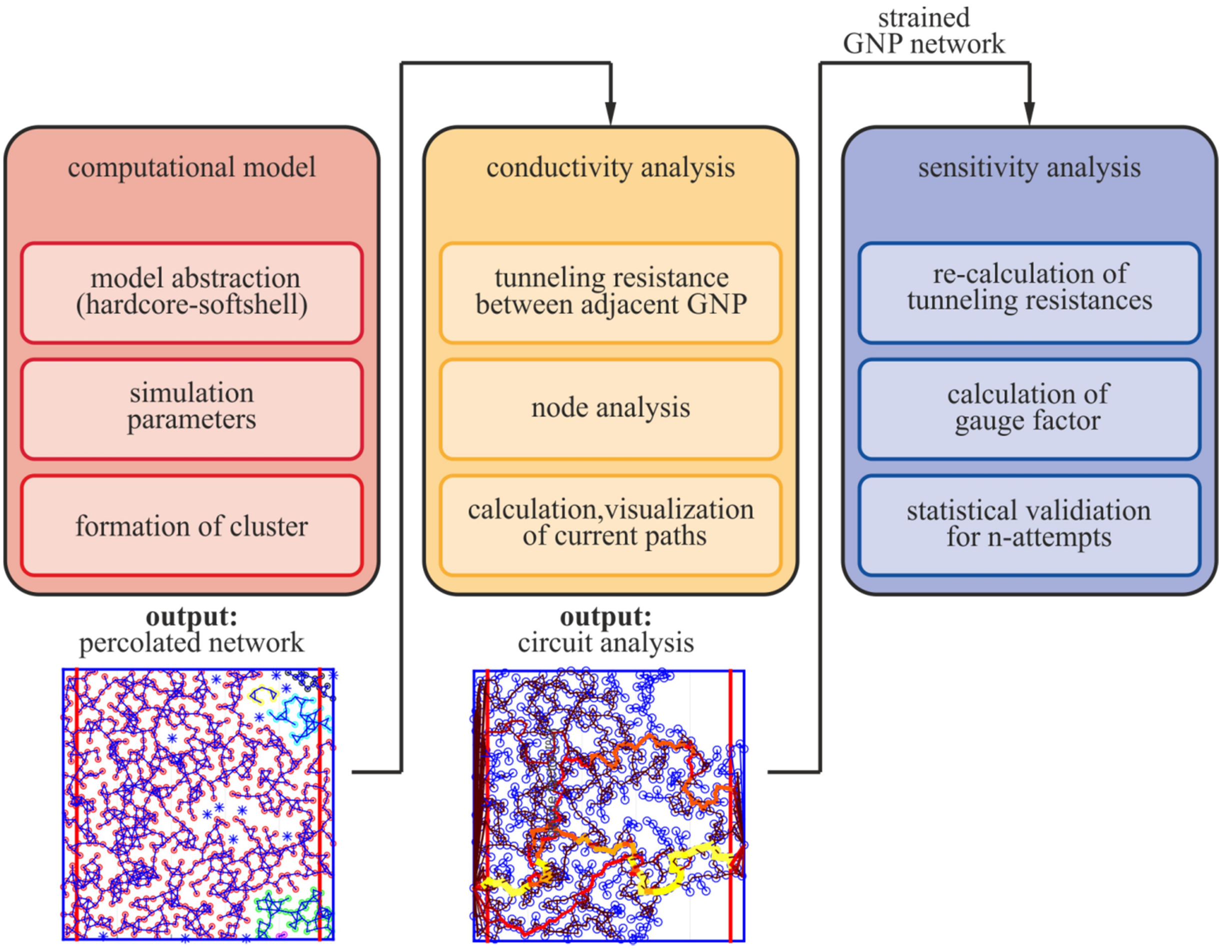

Supplementary Figure S6. Flow chart of our modeling procedure for the 2D or 3D model shown in Section 2.2 of the manuscript. The shown percolated network schematically represents the connected cluster and the corresponding current paths in the 2D model. The current paths are visualized and represented by the line width and colour. Paths with higher current strength are represented with thicker lines in yellow/red colour while paths with small or without any current correspond to blue lines.

Calculation of tunneling junction resistance between i-th and j-th connected GNP given by eq. S8 toS10

$$R_{ij} = \frac{t_{ij} \cdot b}{A} \cdot \exp(t_{ij} \cdot c)\ , \qquad \text{(S8)}$$

$$b = \frac{h^2}{e^2 \sqrt{2m\lambda}}\ , \qquad \text{(S9)}$$

$$c=\frac{4\pi}{h}\sqrt{2m\lambda}\ , \qquad \text{(S10)}$$

where e is the elementary charge, m is the mass of an electron, h is the Planck constant and λ is the potential barrier of the insulator through which the tunneling effect occurs.

Supplementary Section 5. Strain rosette theory.

According to rosette theory, the principal strains ε_1 and ε_2 can be calculated based on three longitudinal strain ε_{L1}, ε_{L2} and ε_{L3}, as given by

$$\varepsilon_{1,2}=\frac{\varepsilon_{L1}+\varepsilon_{L3}}{2}\pm\frac{1}{\sqrt{2}}\sqrt{(\varepsilon_{L1}-\varepsilon_{L2})^2+(\varepsilon_{L2}-\varepsilon_{L3})^2}\ . \quad \text{(S11)}$$

The corresponding principal angle α is given by

$$\alpha=\frac{1}{2}\tan^{-1}\left(\frac{\varepsilon_{L1}-2\varepsilon_{L2}+\varepsilon_{L3}}{\varepsilon_{L1}-\varepsilon_{L3}}\right), \quad \text{(S12)}$$

and describes the angle between the direction of principal strain ε_1 and the longitudinal strain ε_{L1}.
